# Control of Optical Reflection in Ca_2_MgWO_6_ by Co and Mo Doping

**DOI:** 10.3390/molecules29081886

**Published:** 2024-04-21

**Authors:** Kazuki Yamaguchi, Kohei Minagawa, Ryohei Oka, Toshiyuki Masui

**Affiliations:** 1 Department of Chemistry and Biotechnology, Faculty of Engineering, Tottori University, 4-101, Koyama-cho Minami, Tottori 680-8552, Japan; yamaguchi@tottori-u.ac.jp (K.Y.); m20j5040a@gmail.com (K.M.); 2 Center for Research on Green Sustainable Chemistry, Tottori University, 4-101, Koyama-cho Minami, Tottori 680-8552, Japan; 3 Field of Advanced Ceramics, Department of Life Science and Applied Chemistry, Nagoya Institute of Technology, Gokiso, Showa, Nagoya 466-8555, Japan; oka.ryohei@nitech.ac.jp

**Keywords:** bandgap, red pigment, double perovskite, Co^2+^, Mo^6+^

## Abstract

To develop novel inorganic red pigments without harmful elements, we focused on the band structure of Ca_2_(Mg, Co)WO_6_ and attempted to narrow its bandgap by replacing the W^6+^ sites in the host structure of Mo^6+^. Ca_2_Mg_1−*x*_Co*_x_*W_1−*y*_Mo*_y_*O_6_ (0.10 ≤ *x* ≤ 0.30; 0.45 ≤ *y* ≤ 0.60) samples were synthesized by a sol-gel method using citric acids, and the crystal structure, optical properties, and color of the samples were characterized. The Ca_2_Mg_1−*x*_Co*_x_*W_1−*y*_Mo*_y_*O_6_ solid solution was successfully formed, which absorbed visible light at wavelengths below 600 nm. In addition, the absorption wavelength shifted to longer wavelengths with increasing Mo^6+^ content. This is because a new conduction band composed of a Co_3d_-W_5d_-Mo_4d_ hybrid orbital was formed by Mo^6+^ doping to reduce the bandgap energy. Thus, the color of the samples gradually changed from pale orange to dark red, with a hue angle (*h*°) of less than 35°. Based on the above results, the optical absorption wavelength of the Ca_2_Mg_1−*x*_Co*_x_*W_1−*y*_Mo*_y_*O_6_ system can be controlled to change the color by adjusting the bandgap energy.

## 1. Introduction

Inorganic pigments have been utilized for the coloration of ceramics, plastics, glasses, etc., because of their high thermal stability, light resistance, and hiding power. Red inorganic pigments, which indicate warning colors, have been in large demand for applications such as traffic paints. Minium (Pb_3_O_4_·2PbO·PbO_2_), vermilion (HgS), and cadmium red (CdS·CdSe) were used as the red inorganic pigments. Although they exhibit a vivid red color and excellent durability, they contain highly toxic elements, such as Pb, Hg, Cd, and Se, which have negative effects on the human body and environment. Therefore, they are either regulated or banned worldwide. This regulation affects various inorganic materials and is not limited to pigments. For example, toxic HgCl_2_ catalysts have been made to be replaced by mercury-free catalysts [1]. In this context, the development of environmentally friendly inorganic pigments containing less or no toxic elements has been strongly desired to replace existing harmful inorganic pigments.

Recently, sulfides and oxynitride-based pigments such as Ce_2_S_3_ and Ca_1−*x*_La*_x_*TaO_2−*x*_N_1+*x*_ have attracted attention as very brilliant color materials [2,3,4]. However, the chemical stability of sulfide pigments is poor and may cause discoloration when mixed with other pigments. The synthesis of oxynitride pigments requires a prolonged flow of toxic ammonia gas. In addition, sulfur oxide or nitrogen oxide gases are generated during calcination. Thus, oxide pigments are preferred for practical use because they are chemically stable. Although several studies have been reported on oxide pigments [5,6,7,8,9,10,11,12,13], environmentally benign reddish inorganic pigments are not yet comparable to conventionally harmful ones.

In this study, we focus on divalent Co ions (Co^2+^) to develop a novel red inorganic pigment. Octahedrally coordinated Co^2+^ ions are expected to exhibit a red or reddish violet color because they absorb green visible light between 490 and 560 nm based on the d–d transition attributed to ^4^T_1_(F) → ^4^T_1_(P) [14,15,16]. LiCoPO_4_ and (Zn_1−*x*_Co*_x_*)Al_2_O_4_ with Co^2+^ ions have been studied for the development of reddish pigments [15,16], and cobalt violet (Co_3_(PO_4_)_2_) is well known as commercially available. Although there are many materials containing Co for pigments, alloys, catalysis, etc. [17,18,19,20], the Co content tends to be reduced to avoid price escalation because of its high cost. Against this background, there have been many studies on the development of inorganic pigments with low Co contents [21,22,23,24,25,26].

Here, we adopted double-perovskite-type Ca_2_MgWO_6_ as a host material for novel inorganic red pigments. Double-perovskite-type oxides are generally referred to as A_2_BB′O_6_, where A is a cation with a large ionic radius, B and B’ are cations smaller than A, and the total valences of A, B, and B’ are +12. Ca_2_MgWO_6_ has a space group of *P*2_1_/*n* and a monoclinic structure, which consists of 8-coordinate Ca^2+^ at the A site and 6-coordinate octahedral [MgO_6_] and [WO_6_] alternating at the B and B’ sites [27,28]. In addition, Ca_2_MgWO_6_ is a promising phosphor candidate because of its excellent fluorescent properties [27,28,29]; however, few studies have been conducted on this topic.

From the above, we synthesized Ca_2_Mg_1−*x*_Co*_x_*WO_6_ (0 ≤ *x* ≤ 0.50) samples that were partially substituted with Co^2+^ at the Mg^2+^ site of the Ca_2_MgWO_6_ host structure and evaluated their color. Upon substitution with Co^2+^, the samples became light orange, and the color of the samples changed to brown as the Co^2+^ content increased, enhancing the light absorption attributed to the d–d transition of Co^2+^. Among these samples, Ca_2_Mg_1−*x*_Co*_x_*WO_6_ (0.10 ≤ *x* ≤ 0.30) can be a reddish pigment by strengthening the absorbance of the green-light region between 490 and 560 nm while maintaining the reflectance of the red-light region between 605 and 750 nm.

**Figure 1:**
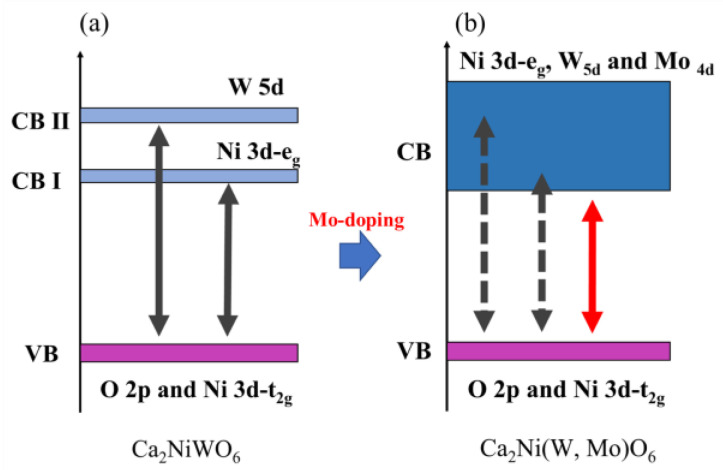
Schematic illustration of band structure for (**a**) Ca_2_NiWO_6_ and (**b**) Ca_2_Ni(W, Mo)O_6_. The black arrows indicate the band gap energy of the host compound, whereas the red arrows indicate the band gap energy of the Mo-doped compound.

For the double-perovskite structure of Ca_2_NiWO_6_, the bandgap energy can be reduced by partially replacing the W^6+^ site with Mo^6+^ [30]. The band structure of Ca_2_NiWO_6_ as shown in Figure 1 has a valence band with an orbital hybridized by the t_2g_ orbital of the Ni_3d_ and O_2p_ orbitals and two conduction bands: the low-energy e_g_ orbital of Ni_3d_ and the high-energy W_5d_ orbital [30,31]. By substituting Mo^6+^ for the W^6+^ site in this compound, a conduction band composed of the Ni_3d_-W_5d_-Mo_4d_ hybrid orbital was newly formed, and the bandgap energy was reduced [30]. In this study, we attempted to replace the W^6+^ site in the host structure with Mo^6+^ to narrow the bandgap and exert a red color by expanding the light absorption wavelength to longer wavelengths. In other words, the Ca_2_Mg_1−*x*_Co*_x_*W_1−*y*_Mo*_y_*O_6_ (0.10 ≤ *x* ≤ 0.30; 0.45 ≤ *y* ≤ 0.60) samples were synthesized, and their color was evaluated. 

## 2. Results and Discussion

### 2.1. Ca_2_Mg_1−x_Co_x_WO_6_ (0 ≤ x ≤ 0.50)

#### 2.1.1. X-ray Powder Diffraction

**Figure 2:**
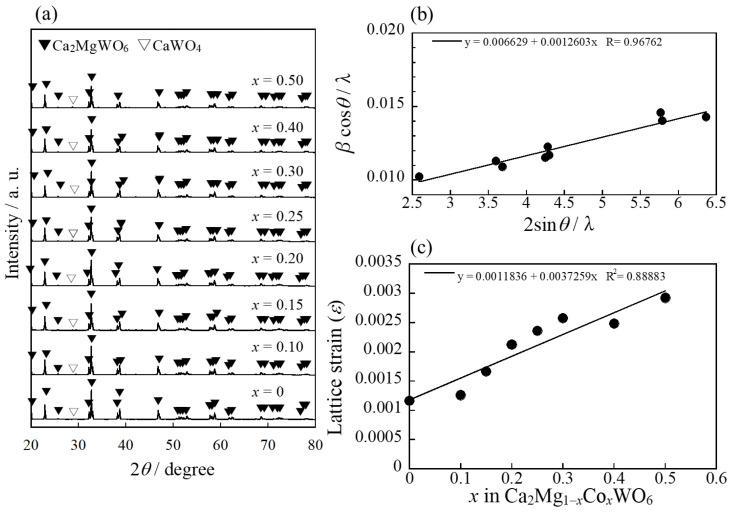
(**a**) XRD pattern of the Ca_2_Mg_1−_*_x_*Co*_x_*WO_6_ (0 ≤ *x* ≤ 0.50) samples; (**b**) Williamson-Hall (W-H) plot of Ca_2_MgWO_6_; and (**c**) composition dependence of lattice strain (*ε*) calculated by W-H analysis.

Figure 2a shows the X-ray powder diffraction (XRD) patterns of the Ca_2_Mg_1−*x*_Co*_x_*WO_6_ (0 ≤ *x* ≤ 0.50) samples. The Ca_2_MgWO_6_ phase was obtained for all samples as the main phase, but a few peaks of CaWO_4_ were observed as an impurity phase.

To investigate the lattice strain of these samples, a Williamson-Hall (W-H) analysis was conducted following the equation *β*cos*θ*/*λ =* 2*ε*sin*θ*/*λ + K/D*, where *K* = 0.94 and *β*, *θ*, *λ*, *D*, and *ε* represent the peak width at half-maximum intensity, diffraction position, wavelength of radiation, crystallite size, and strain component, respectively [32,33]. The W-H plot of Ca_2_MgWO_6_ is shown in Figure 2b, where the slope and intercept represent the strain component and crystallite size, respectively. The lattice strain of all the samples was also estimated using the W-H plot and is summarized in Figure 2c. The lattice strain of the samples increased with increasing Co content, indicating that Co^2+^ was partially introduced into the host lattice. Therefore, the probability of the d–d transition of Co^2+^ should increase with the Co concentration in these systems.

**Figure 3:**
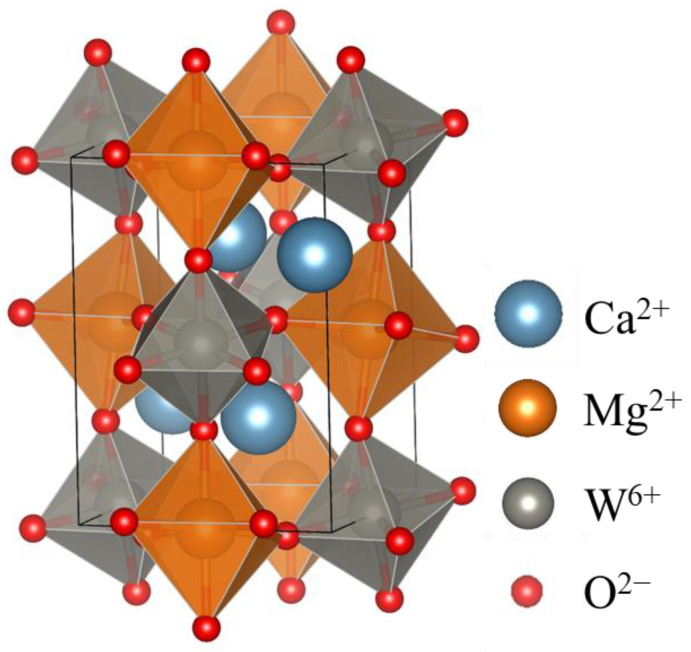
Crystal structure of double-perovskite Ca_2_MgWO_6_; Ca: blue, Mg: orange, W: gray, O: red.

**Figure 4:**
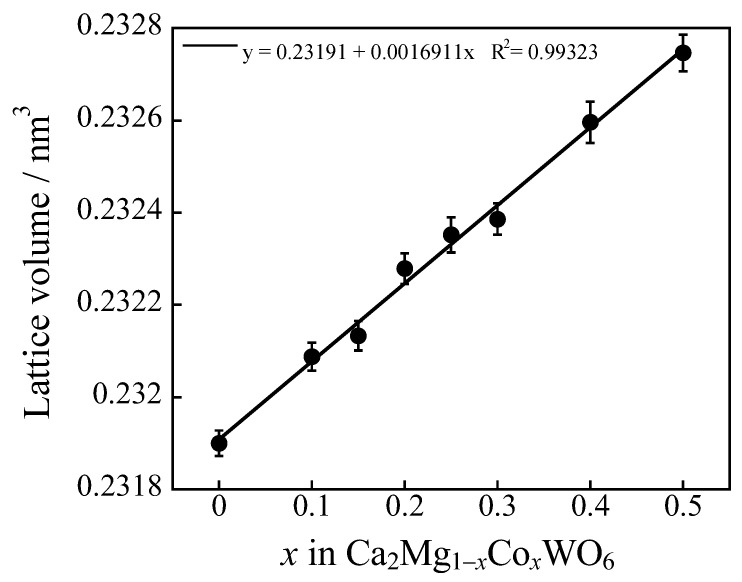
Composition dependence of the lattice volume of the Ca_2_Mg_1−_*_x_*Co*_x_*WO_6_ (0 ≤ *x* ≤ 0.50) samples.

The Ca_2_MgWO_6_ double-perovskite structure was illustrated by the VESTA program [34], as shown in Figure 3. It has octahedral Mg^2+^ sites that can be partially replaced by Co^2+^ ions. The composition dependence of the lattice volume of the samples, calculated from each XRD pattern, is shown in Figure 4. The lattice volume of the samples increased monotonically with good linearity (determination coefficient: R^2^ > 0.99) by increasing the Co^2+^ content in the 0 ≤ *x* ≤ 0.50 range. This phenomenon means that larger Co^2+^ (ionic radius: 0.0745 nm, 6-coordination site [35]) ions were partially introduced into the Mg^2+^ (ionic radius: 0.072 nm, 6-coordination site [35]) sites of the host structure. Thus, Ca_2_Mg_1−*x*_Co*_x_*WO_6_ (0 ≤ *x* ≤ 0.50) solid solutions were successfully synthesized.

#### 2.1.2. Ultraviolet–Visible Reflectance Spectra

**Figure 5:**
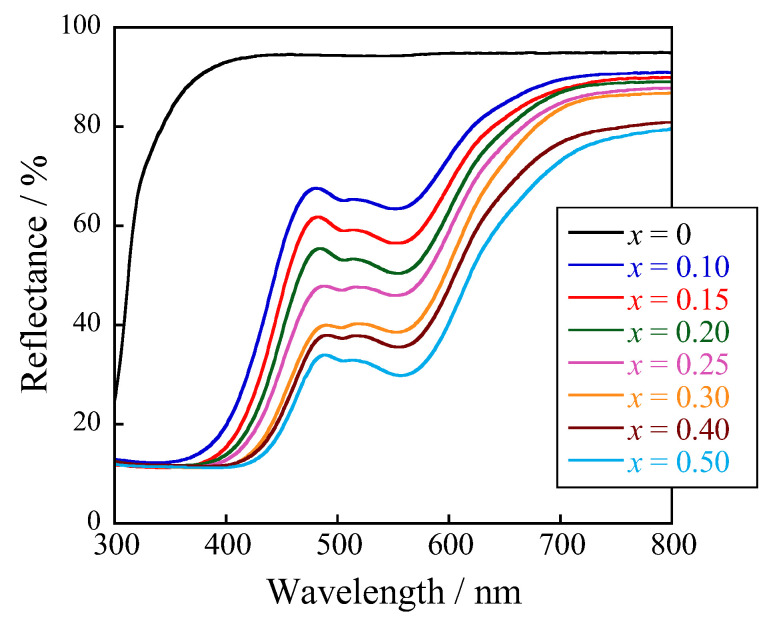
UV–vis reflectance spectra of the Ca_2_Mg_1−*x*_Co*_x_*WO_6_ (0 ≤ *x* ≤ 0.50) samples.

The optical properties of the Ca_2_Mg_1−*x*_Co*_x_*WO_6_ (0 ≤ *x* ≤ 0.50) samples were evaluated by ultraviolet–visible (UV–vis) reflectance spectroscopy. The UV–vis reflectance spectra of the samples are shown in Figure 5. Ca_2_MgWO_6_ (*x* = 0), as a host compound, strongly reflected all visible light regions. In contrast, the samples with Co^2+^ absorbed visible light at wavelengths of 350 nm and between 500 and 600 nm. The former light absorption at approximately 350 nm was attributed to the ligand-to-metal charge transfer (LMCT) transition of O^2—^ to Co^2+^ [36], and the latter between 500 and 600 nm was assigned to the d–d transition (^4^T_1_(F) → ^4^T_1_(P)) of Co^2+^ [14,15,16]. The absorption wavelength for the LMCT transition of Co^2+^ shifted to longer wavelengths with the Co^2+^ content owing to lattice volume expansion, leading to longer Co–O bond lengths. In addition, the optical absorption based on the d–d transition was enhanced with increasing Co^2+^ concentration, as shown in Figure 2c. This d–d transition (^4^T_1_(F) → ^4^T_1_(P)) is a spin-allowed transition, and the transition probability increases with not only the Co^2+^ content but also the lattice strain.

#### 2.1.3. Color Property

**Figure 6:**
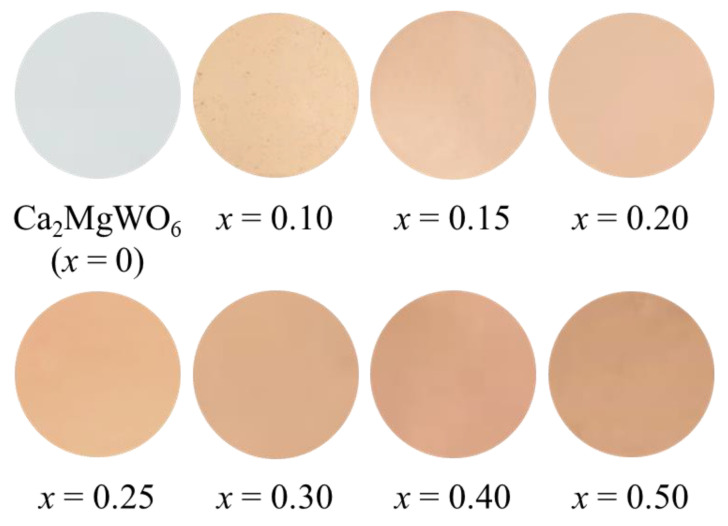
Photographs of the Ca_2_Mg_1−*x*_Co*_x_*WO_6_ (0 ≤ *x* ≤ 0.50) powder samples.

The L*a*b*Ch° color coordinate data for the Ca_2_Mg_1−*x*_Co*_x_*WO_6_ (0 ≤ *x* ≤ 0.50) powder samples are listed in Table 1. Photographs of the samples are shown in Figure 6. In the case of *x* = 0, the sample strongly reflected visible light and exhibited a white color. In contrast, for *x* ranging from 0.10 to 0.50, light absorption in the green light region around 490–560 nm increased with increasing Co content. Since the brightness (L*) decreased and the redness (a*) as well as the yellowness (b*) increased, the sample color showed a pale orange for *x* ranging from 0.10 to 0.30 and brown for *x* ranging over 0.40. Thus, it was found that a reddish pigment could not be realized by doping the host lattice with only Co.

### 2.2. Ca_2_Mg_1−x_Co_x_W_1−y_Mo_y_O_6_ (0.10 ≤ x ≤ 0.30; 0.45 ≤ y ≤ 0.60)

#### 2.2.1. X-ray Powder Diffraction

The Co^2+^-doped samples, Ca_2_Mg_1−*x*_Co*_x_*WO_6_ (0.10 ≤ *x* ≤ 0.50), exhibited a pale orange or brown color, and reddish pigments were not obtained. To improve their color, Ca_2_Mg_1−*x*_Co*_x_*W_1−*y*_Mo*_y_*O_6_ (0.10 ≤ *x* ≤ 0.30; 0.45 ≤ *y* ≤ 0.60), in which the W^6+^ site of pale orange Ca_2_Mg_1−*x*_Co*_x_*WO_6_ was partially replaced by Mo^6+^, was synthesized and characterized. These samples are hereafter referred to as Co*a*Mo*b*, where *a* and *b* are the Co and Mo content, respectively. (For example, when Co content is 10% and Mo content is 50%, the sample is described as Co10Mo50.)

**Figure 7:**
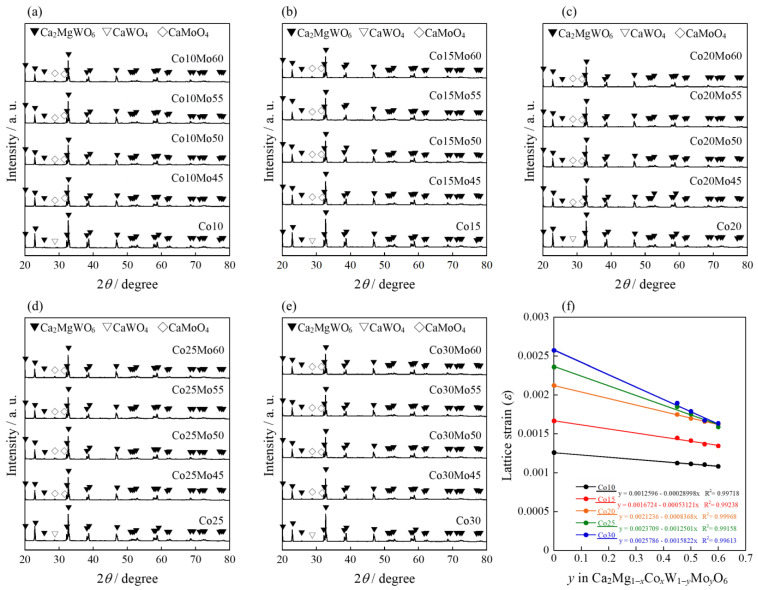
XRD patterns of the Co*a*Mo*b* samples: (**a**) *a* = 10; (**b**) *a* = 15; (**c**) *a* = 20; (**d**) *a* = 25; and (**e**) *a* = 30. (**f**) Composition dependence of lattice strain (*ε*) for Co*a*Mo*b* calculated by W_−_H analysis.

The XRD patterns of the Co*a*Mo*b* powder samples are shown in Figure 7a–e. For comparison, the XRD patterns of the Co*a*Mo0 samples are also shown. Although the target Ca_2_MgWO_6_ phase was obtained as the main phase for all samples, a few peaks of CaMoO_4_ were detected as impurities, resulting in a mixed phase. The lattice strain of all the samples was estimated using a W-H plot, as summarized in Figure 7f. The lattice strain of the samples decreased with increasing Mo content, indicating that Mo^6+^ was partially introduced into the host lattice.

**Figure 8:**
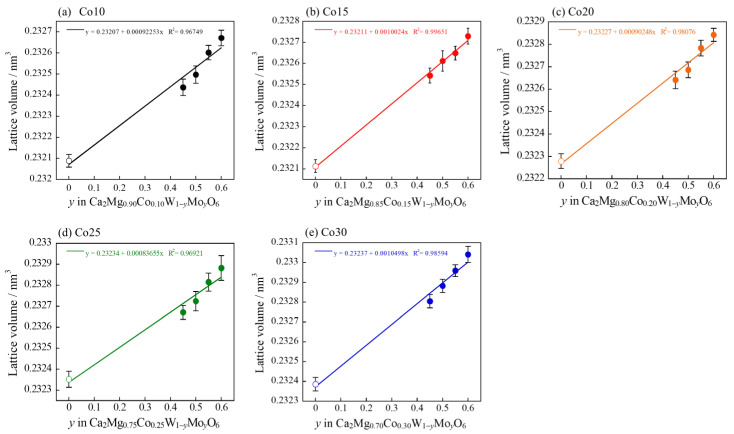
Composition dependence of the lattice volume of the Co*a*Mo*b* samples: (**a**) *a* = 10; (**b**) *a* = 15; (**c**) *a* = 20; (**d**) *a* = 25; and (**e**) *a* = 30.

The dependence of the lattice volume on the composition of all the samples calculated from each XRD pattern is shown in Figure 8. The introduction of smaller Mo^6+^ ions (ionic radius: 0.0590 nm at the 6-coordination site [35]) into the W^6+^ sites (ionic radius: 0.060 nm at the 6-coordination site [35]) should result in a decrease in lattice volume with increasing Mo^6+^ content. However, the lattice volume of the Co*a*Mo*b* samples increased linearly. This is because W^6+^, which has a higher electronegativity (6-coordination: 2.175 [37]) in the Ca_2_(Mg, Co)WO_6_ structure, was partially replaced by Mo^6+^ with less electronegativity (6-coordination: 2.101 [37]), extending the bond length for W/Mo–O to expand the lattice volume. Thus, the synthesized samples successfully formed solid solutions.

#### 2.2.2. UV–Vis Reflectance Spectra

**Figure 9:**
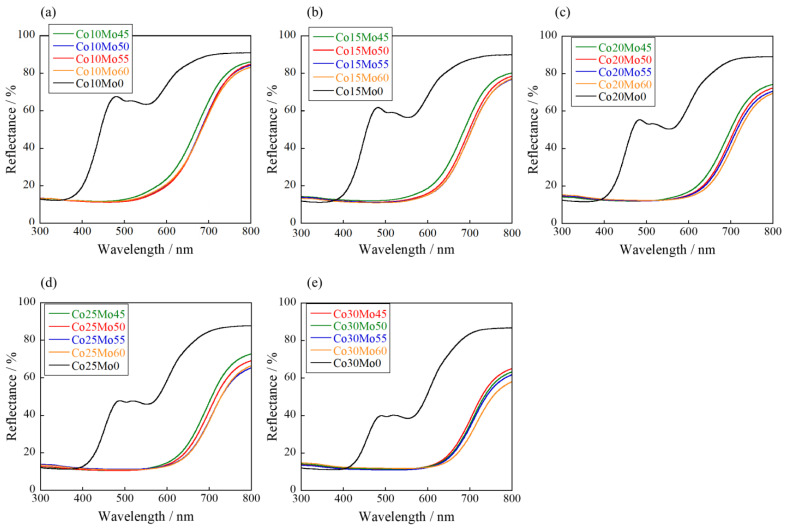
UV–vis reflectance spectra of the Co*a*Mo*b* samples: (**a**) *a* = 10; (**b**) *a* = 15; (**c**) *a* = 20; (**d**) *a* = 25; and (**e**) *a* = 30.

**Figure 10:**
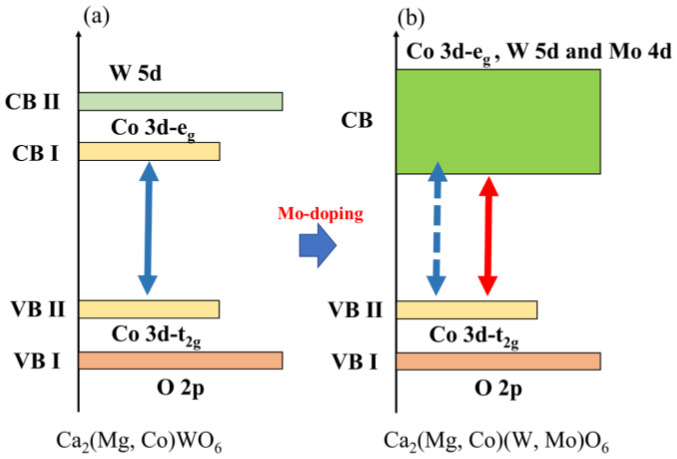
Schematic illustration of band structure for (**a**) Ca_2_(Mg, Co)WO_6_ and (**b**) Ca_2_(Mg, Co(W, Mo)O_6_. The blue arrows indicate the band gap energy of the host compound, whereas the red arrows indicate the band gap energy of the Mo-doped compound.

The UV–Vis reflectance spectra of Co*a*Mo*b* are shown in Figure 9. The results for the Co*a*Mo0 (10 ≤ *a* ≤ 30) samples are shown for comparison. The Mo-doped samples absorbed intensely visible light at wavelengths below 600 nm, and the absorption wavelength shifted to longer wavelengths with increasing Mo concentrations. This is due to the formation of a new conduction band of the Co_3d_-W_5d_-Mo_4d_ hybrid orbital by Mo^6+^ doping [30], which results in a reduction in the bandgap energy, as shown in Figure 10.

**Figure 11:**
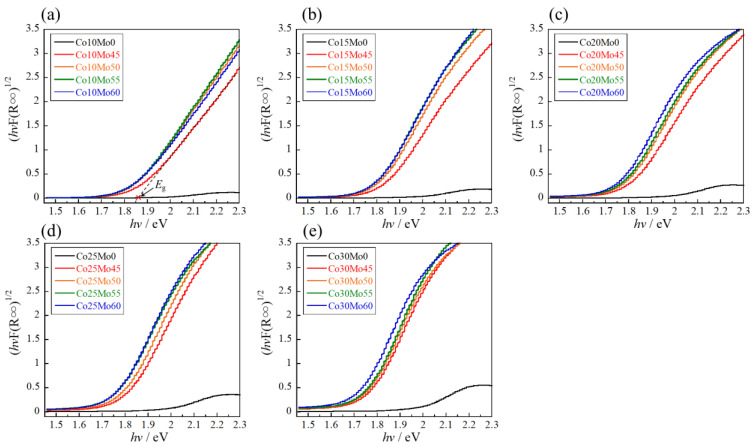
Tauc plot of the Co*a*Mo*b* samples: (**a**) *a* = 10; (**b**) *a* = 15; (**c**) *a* = 20; (**d**) *a* = 25; and (**e**) *a* = 30.

The bandgap energy (*E*_g_) of these samples was investigated using a Tauc plot [38], which was obtained by converting the corresponding UV–vis reflectance spectra, as shown in Figure 11. The bandgap energy was determined from the Tauc plot by extrapolating the linear area across the *hν* axis of the graph. The intersection with the *hν* axis is an estimation of the corresponding *E*_g_. The estimated *E*_g_ values are listed in Table 2. The *E*_g_ values of the Co- and Mo-doped samples were smaller than those of the Co-doped samples, which means that the Co_3d_-W_5d_-Mo_4d_ hybrid orbital as a conduction band was newly constructed by Mo^6+^ doping, as shown in Figure 10. As expected, the optical bandgap energy decreased with increasing Mo content when the Co content was fixed. Therefore, the bandgap energy of the sample could be finely controlled by co-doping with various concentrations of Co and Mo. In addition, the reduction in bandgap energy leads to a higher electrical conductivity of the materials. The samples synthesized using this bandgap-controlling strategy have potential applications in other fields, such as catalysts for water splitting [39].

#### 2.2.3. Color Property

**Figure 12:**
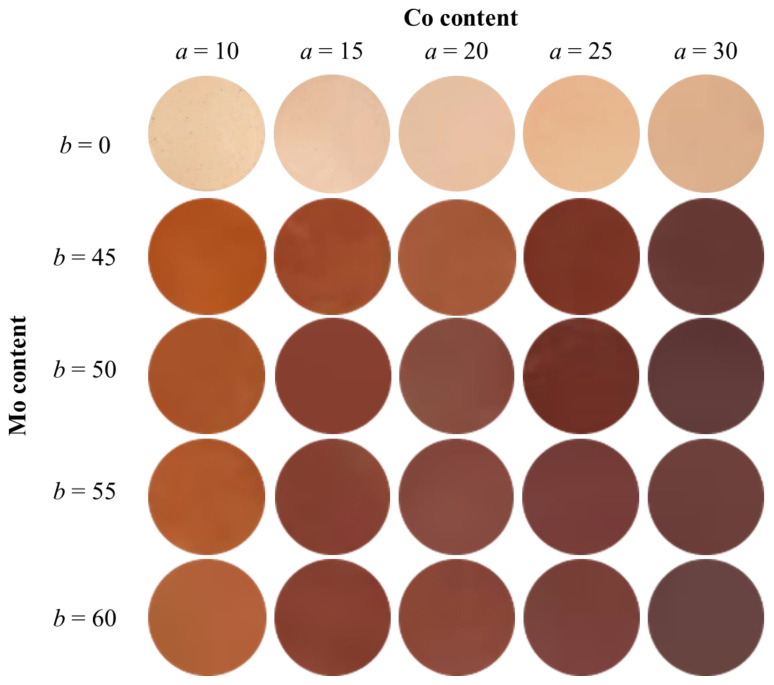
Photographs of the Co*a*Mo*b* samples.

The color properties of the Co*a*Mo*b* samples were evaluated using a colorimeter. The L*a*b*Ch° color coordinates and photographs are presented in Table 3 and Figure 12, respectively. Brightness (L*), redness (a*), and yellowness (b*) decreased with increasing Mo^6+^ content. This is because the samples absorbed not only the green light region (490–560 nm) of the complementary color against the red but also the yellow to red light regions (580–750 nm), as shown in Figure 9. However, the hue angle (*h*°) decreased with increasing Co and Mo content. When the Co concentration was greater than 15%, *h°* ranged from 0 to 35, indicating a red color. In fact, the sample color changed from reddish-brown to dark red upon Mo doping. In addition, the Co15Mo50 samples exhibited the highest a* value among the samples with a red hue angle (0 ≤ *h*° ≤ 35) to be the most nearly red color. Consequently, the color of the sample could be gradually controlled by Co and Mo doping.

**Figure 13:**
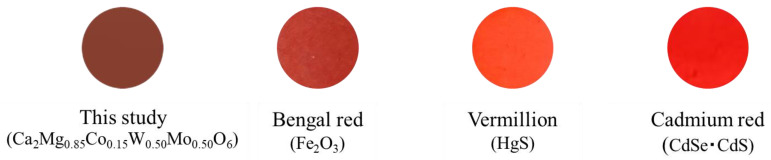
Photographs of Ca_2_Mg_0.85_Co_0.15_W_0.50_Mo_0.50_O_6_, Bengal red, vermillion, and cadmium red pellets made from powder samples.

The chromatic parameters of the Co*a*Mo*b* sample were compared with those of commercially available red pigments such as Bengal red (Fe_2_O_3_), vermillion (HgS), and cadmium red (CdS·CdSe), as listed in Table 4. The photographs are shown in Figure 13. Although the values of a* and b* for Co15Mo50 were smaller than those for commercial red pigments, the pigment synthesized in this study showed the lowest hue angle (*h°* = 33.9), which means that its color is close to the purest red color among these red pigments. However, further improvements are necessary to make them comparable to the red color of conventionally harmful pigments.

#### 2.2.4. Chemical Stability Test

**Figure 14:**
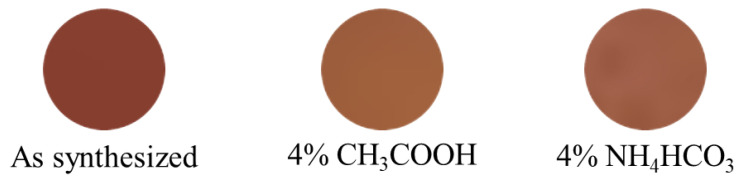
Photographs of Ca_2_Mg_0.85_Co_0.15_W_0.50_Mo_0.50_O_6_ samples before and after the chemical stability test.

If pigments are practically used for various applications, such as tableware, their acid-base resistance is an important property. The chemical stability of the Co15Mo50 powder samples was evaluated. The powder samples were soaked for 7 h at room temperature in 4% CH_3_COOH and 4% NH_4_HCO_3_ aqueous solutions, assuming vinegar and baking soda, which are possibly the acids and bases most likely to come into contact with tableware. The samples were washed with deionized water and ethanol and dried at ambient temperature for 24 h. Table 5 summarizes the chromatic parameters of the samples after acid and base resistance tests, and the corresponding photographs of the samples are shown in Figure 14. Unfortunately, the Co15Mo50 pigment was less chemically stable because the color tone changed after the leaching tests in the acid and base solutions. To suppress color degradation, it is necessary to protect the surface with inert substances such as silica.

## 3. Materials and Methods

### 3.1. Synthesis

Ca_2_Mg_1−*x*_Co*_x_*WO_6_ (0 ≤ *x* ≤ 0.50) samples were synthesized using a citrate sol-gel method. The starting materials were Ca(NO_3_)_2_·4H_2_O (FUJIFILM Wako Pure Chemical Industries Ltd., Osaka, Japan, 98.5%), Mg(NO_3_)_2_·6H_2_O (FUJIFILM Wako Pure Chemical Industries Ltd., 99.5%), Co(NO_3_)_2_·6H_2_O (FUJIFILM Wako Pure Chemical Industries Ltd., 99.5%), and WO_3_ (Kishida Chemical Co. Ltd., Osaka, Japan, 98.0%). The materials were weighed stoichiometrically to obtain the desired compositions, as listed in Table 6. WO_3_ was dissolved in 30 cm^3^ of five-fold diluted aqueous ammonia (FUJIFILM Wako Pure Chemical Industries Ltd., 28.0 wt. %), and the metal nitrates were dissolved in 50 cm^3^ of deionized water. These solutions were mixed and stirred uniformly, and citric acid (CA; FUJIFILM Wako Pure Chemical Industries Ltd., 98.0%) was added as a chelator to complex the cations in the solution. The molar ratio of the total cations (Ca, Mg, Co, and W) to CA was 2:1. The mixed solution was stirred by heating at 80 °C until a gel was obtained, which was then oven-dried at 120 °C for 24 h. The dried gel was pulverized using an agate mortar and calcined in an alumina crucible at 500 °C for 8 h in air. After calcination, the sample was again heated in an alumina boat at 1250 °C for 5 h in air. The samples were ground in an agate mortar and pestle before characterization.

Co*a*Mo*b* (10 ≤ *a* ≤ 30; 45 ≤ *b*≤ 60), namely Ca_2_Mg_1−*x*_Co*_x_*W_1−*y*_Mo*_y_*O_6_ (0.10 ≤ *x* ≤ 0.30; 0.45 ≤ *y* ≤ 0.60), samples were also prepared using a procedure similar to that described above. The starting materials, Ca(NO_3_)_2_·4H_2_O, Mg(NO_3_)_2_·6H_2_O, Co(NO_3_)_2_·6H_2_O, MoO_3_ (FUJIFILM Wako Pure Chemical Industries Ltd., 99.9%), and WO_3_, were stoichiometrically weighed, as shown in Table 7. MoO_3_ and WO_3_ were dissolved in 30 cm^3^ of five-fold diluted aqueous ammonia.

### 3.2. Characterization

Powder X-ray diffraction (XRD) analysis was performed using an Ultima IV X-ray diffractometer (Rigaku Corporation, Tokyo, Japan) to identify the crystal phases and structures of the samples. XRD patterns were obtained using Cu-Kα radiation, which was operated at a tube voltage of 40 kV and a tube current of 40 mA. The data were collected by scanning over a 2*θ* range of 20°–80°. The sampling width was 0.02°, and the scan speed was 6° min^−1^. The lattice volumes were calculated from the XRD peak angles refined by α-Al_2_O_3_ as a standard and using CellCalc Ver. 2.20 software. An ultraviolet–visible (UV–Vis) spectrometer (JASCO Corporation, Tokyo, Japan, V-770 with an integrating sphere attachment) was used to record the optical reflectance spectra of the as-prepared samples using a standard white plate as a reference. The step width was 1 nm, and the scan rate was 1000 nm min^−1^. The bandgap energies of the samples were calculated from the absorption edge of the absorbance spectrum, represented by the Kubelka-Munk function *f*(R) = (1 − R)^2^/2R, where *f*(R) is the theoretical absorbance and R is the measured reflectance [36]. The chromatic parameters of the powder samples were evaluated based on the Commission Internationale de l’Éclairage (CIE) L*a*b*Ch° system using a colorimeter (Konica-Minolta, Inc., Tokyo, Japan, CR-400). A standard C illuminant was used for colorimetric measurements. The L* parameter shows the brightness or darkness in neutral grayscale. Positive and negative a* values represent reddish and greenish colors, respectively. Positive and negative b* values indicate yellowish and bluish colors, respectively. The chroma parameter (*C*) is the color saturation, which is expressed by the formula C = [(a*)^2^ + (b*)^2^]^1/2^. The hue angle (*h*°) ranged from 0° to 360° and was calculated using the equation *h*° = arctan(b*/a*). For the L*a*b*Ch° color coordinate data, all values showed standard deviations of less than 0.1.

## 4. Conclusions

To develop novel inorganic red pigments with fewer harmful elements, we focused on double-perovskite-type Ca_2_MgWO_6_ and Co^2+^ ions as the host material and chromophores, respectively. Ca_2_Mg_1−*x*_Co*_x_*WO_6_ (0 ≤ *x* ≤ 0.50) samples were synthesized using the citrate sol-gel method. The sample color turned from white to brown with an increase in Co^2+^ content due to enhanced light absorption based on the d–d transition, but a reddish color was not obtained. Thus, we attempted to lower the bandgap energy of Ca_2_Mg_1−*x*_Co*_x_*WO_6_ to achieve a red color, and the Co*a*Mo*b* (10 ≤ *a* ≤ 30; 45 ≤ *b* ≤ 60) samples were prepared and characterized. When Mo^6+^ was introduced into the W^6+^ site of Ca_2_Mg_1−*x*_Co*_x_*WO_6_, a new conduction band corresponding to the Co_3d_-W_5d_-Mo_4d_ hybridized orbital was formed, and their bandgap energies decreased, as expected. The bandgap energy decreased with increasing Mo^6+^ concentrations because of the widening conduction band. As a result, the samples absorbed longer wavelengths of light and exhibited a reddish-brown or dark-red color. Among the samples whose hue angle ranged in red (0 ≤ *h*° ≤ 35), Co15Mo50 showed the highest a* value of 22.3. Although the vividness of the sample synthesized in this study was less than that of commercially available inorganic red pigments, its chromatic purity was the highest among them. It is noteworthy that the bandgap energy can be controlled by introducing the Mo_4d_ orbital between the Co_3d_ and W_4d_ orbitals to form a new wide conduction band; thus, the color of the sample is also controllable.

## Figures and Tables

**Table 1:** Color coordinates of the Ca_2_Mg_1−*x*_Co*_x_*WO_6_ (0 ≤ *x* ≤ 0.50) samples.

*x*	L*	a*	b*	*C*	*h*°
0	96.4	+0.70	−0.08	0.70	353
0.10	86.5	+5.05	+12.3	13.3	67.7
0.15	83.1	+6.12	+14.9	16.1	67.7
0.20	78.8	+8.24	+18.2	20.0	65.6
0.25	75.0	+9.57	+22.3	24.3	66.8
0.30	71.0	+12.3	+25.6	28.4	64.3
0.40	67.0	+11.4	+25.0	27.7	65.5
0.50	62.7	+12.3	+23.0	26.0	61.9

**Table 2:** Bandgap energy (*E*_g_) for the Co*a*Mo*b* samples estimated from the UV–vis reflectance spectra.

Samples	*E*_g_/eV
Co10Mo0	1.99
Co10Mo45	1.86
Co10Mo50	1.84
Co10Mo55	1.83
Co10Mo60	1.82
Co15Mo0	1.99
Co15Mo45	1.81
Co15Mo50	1.80
Co15Mo55	1.79
Co15Mo60	1.78
Co20Mo0	1.97
Co20Mo45	1.80
Co20Mo50	1.78
Co20Mo55	1.77
Co20Mo60	1.75
Co25Mo0	1.97
Co25Mo45	1.79
Co25Mo50	1.78
Co25Mo55	1.75
Co25Mo60	1.74
Co30Mo0	1.97
Co30Mo45	1.77
Co30Mo50	1.75
Co30Mo55	1.74
Co30Mo60	1.73

**Table 3:** Color coordinates for the Co*a*Mo*b* samples.

Sample	L*	a*	b*	*C*	*h*°
Co10Mo0	86.5	+5.05	+12.3	13.3	67.7
Co10Mo45	40.4	+26.3	+31.3	40.9	50.0
Co10Mo50	36.8	+24.8	+23.6	34.2	43.6
Co10Mo55	36.0	+25.8	+24.0	35.2	42.9
Co10Mo60	37.2	+24.5	+24.2	34.4	44.6
Co15Mo0	83.1	+6.12	+14.9	16.1	67.7
Co15Mo45	35.0	+24.8	+20.7	32.3	39.9
Co15Mo50	31.2	+22.3	+15.0	26.9	33.9
Co15Mo55	31.1	+22.1	+14.6	26.5	33.5
Co15Mo60	29.8	+21.6	+13.4	25.4	31.8
Co20Mo0	78.8	+8.24	+18.2	20.0	65.6
Co20Mo45	31.0	+22.9	+17.1	28.6	36.7
Co20Mo50	30.0	+20.2	+11.7	23.3	30.1
Co20Mo55	27.9	+19.2	+10.6	21.9	28.9
Co20Mo60	28.2	+19.0	+10.3	21.6	28.5
Co25Mo0	75.0	+9.57	+22.3	24.3	66.8
Co25Mo45	29.7	+21.9	+13.7	25.8	32.0
Co25Mo50	26.3	+20.2	+11.8	23.4	30.3
Co25Mo55	25.4	+15.9	+7.53	17.6	25.3
Co25Mo60	25.7	+16.3	+7.64	18.0	25.1
Co30Mo0	71.0	+12.3	+25.6	28.4	64.3
Co30Mo45	27.0	+16.0	+6.86	17.4	23.2
Co30Mo50	25.4	+14.4	+5.46	15.4	20.8
Co30Mo55	25.5	+14.3	+5.54	15.3	21.2
Co30Mo60	25.4	+10.8	+3.24	11.3	16.7

**Table 4:** Color coordinates of various red pigments.

Samples	L*	a*	b*	*h*°
Co15Mo50	31.2	+22.3	+15.0	33.9
Bengal red (Fe_2_O_3_)	36.7	+33.1	+25.0	37.0
Vermillion (HgS)	53.5	+55.6	+42.9	37.7
Cadmium red (CdS·CdSe)	54.0	+61.8	+55.3	41.8

**Table 5:** Color coordinate data of the Co15Mo50 samples before and after the chemical stability test.

Treatment	L*	a*	b*	*C*	*h*°
As synthesized	31.2	+22.3	+15.0	26.9	33.9
4% CH_3_COOH	39.8	+22.0	+21.6	30.8	44.5
4% NH_4_HCO_3_	40.2	+20.2	+22.0	28.4	44.7

**Table 6:** Amounts of reagents used to synthesize Ca_2_Mg_1−*x*_Co*_x_*WO_6_ (0 ≤ *x* ≤ 0.50).

Sample	Ca(NO_3_)_2_·4H_2_O	Mg(NO_3_)_2_·6H_2_O	Co(NO_3_)_2_·6H_2_O	WO_3_	CA
Ca_2_MgWO_6_	1.2290 g	0.6672 g	-	0.6033 g	3.9994 g
Co10	1.2180 g	0.5951 g	0.0751 g	0.5979 g	3.9637 g
Co15	1.2126 g	0.5296 g	0.1121 g	0.5952 g	3.9460 g
Co20	1.2072 g	0.5243 g	0.1488 g	0.5926 g	3.9286 g
Co25	1.2019 g	0.4894 g	0.1852 g	0.5900 g	3.9113 g
Co30	1.1966 g	0.4548 g	0.2212 g	0.5874 g	3.8941 g
Co40	1.1862 g	0.3864 g	0.2924 g	0.5823 g	3.8602 g
Co50	1.1760 g	0.3192 g	0.3623 g	0.5773 g	3.8269 g

**Table 7:** Amounts of reagents used to synthesize Co*a*Mo*b* (10 ≤ *a* ≤ 30; 45 ≤ *b*≤ 60).

Sample	Ca(NO_3_)_2_·4H_2_O	Mg(NO_3_)_2_·6H_2_O	Co(NO_3_)_2_·6H_2_O	WO_3_	MoO_3_	CA
Co10Mo45	1.3564 g	0.6627 g	0.0836 g	0.3662 g	0.1860 g	4.4139 g
Co10Mo50	1.3737 g	0.6712 g	0.0846 g	0.3372 g	0.2093 g	4.4703 g
Co10Mo55	1.3915 g	0.6799 g	0.0857 g	0.3074 g	0.2333 g	4.5281 g
Co10Mo60	1.4097 g	0.6888 g	0.0869 g	0.2768 g	0.2578 g	4.5875 g
Co15Mo45	1.3496 g	0.6228 g	0.1247 g	0.3644 g	0.1851 g	4.3920 g
Co15Mo50	1.3668 g	0.6338 g	0.1263 g	0.3355 g	0.2083 g	4.4479 g
Co15Mo55	1.3844 g	0.6388 g	0.1280 g	0.3058 g	0.2321 g	4.5051 g
Co15Mo60	1.4025 g	0.6472 g	0.1296 g	0.2754 g	0.2565 g	4.5640 g
Co20Mo45	1.3430 g	0.5833 g	0.1655 g	0.3626 g	0.1842 g	4.3704 g
Co20Mo50	1.3600 g	0.5907 g	0.1676 g	0.3338 g	0.2073 g	4.4257 g
Co20Mo55	1.3774 g	0.5982 g	0.1698 g	0.3043 g	0.2309 g	4.4824 g
Co20Mo60	1.3953 g	0.6060 g	0.1720 g	0.2740 g	0.2552 g	4.5406 g
Co25Mo45	1.3364 g	0.5442 g	0.2059 g	0.3607 g	0.1833 g	4.3490 g
Co25Mo50	1.3532 g	0.5510 g	0.2085 g	0.3321 g	0.2062 g	4.4037 g
Co25Mo55	1.3705 g	0.5580 g	0.2111 g	0.3027 g	0.2297 g	4.4599 g
Co25Mo60	1.3882 g	0.5652 g	0.2139 g	0.2726 g	0.2539 g	4.5175 g
Co30Mo45	1.3299 g	0.5054 g	0.2458 g	0.3590 g	0.1824 g	4.3278 g
Co30Mo50	1.3466 g	0.5117 g	0.2489 g	0.3305 g	0.2052 g	4.3820 g
Co30Mo55	1.3637 g	0.5182 g	0.2521 g	0.3012 g	0.2286 g	4.4376 g
Co30Mo60	1.3812 g	0.5249 g	0.2553 g	0.2712 g	0.2526 g	4.4946 g

## Data Availability

Data are contained within this article.
